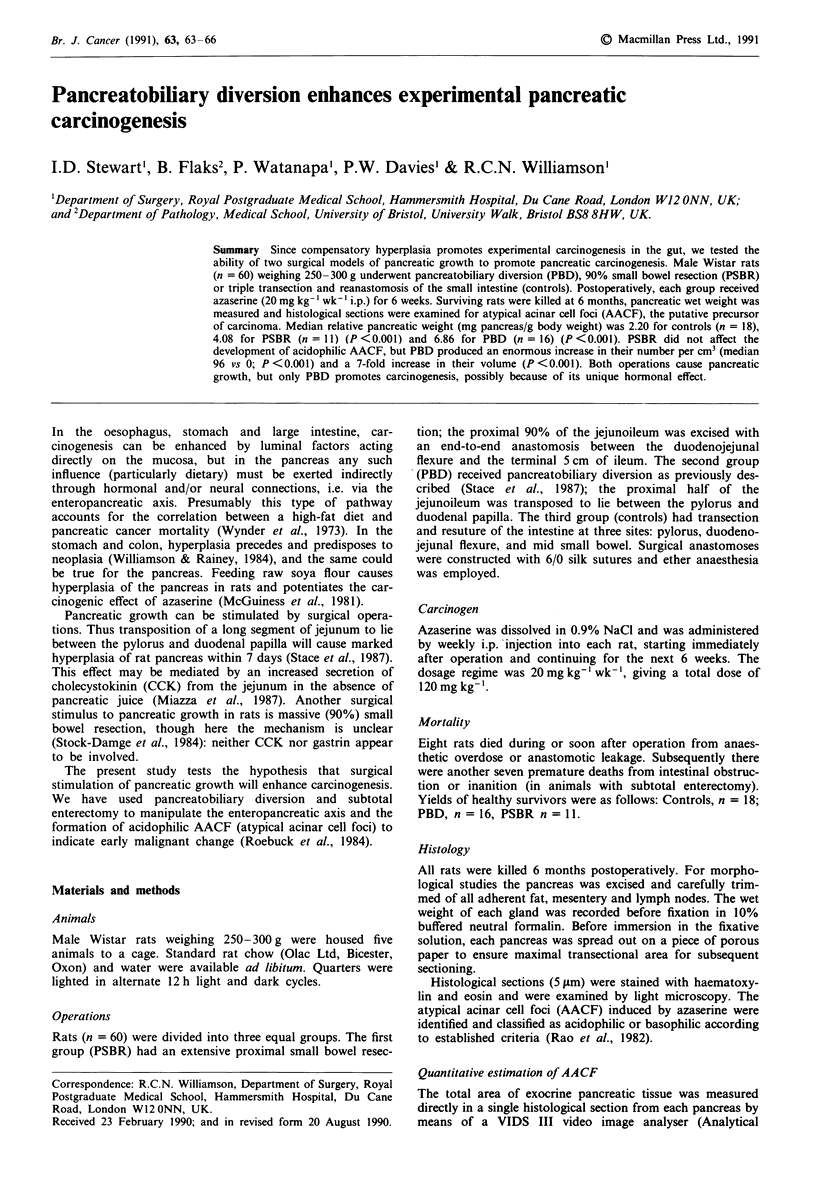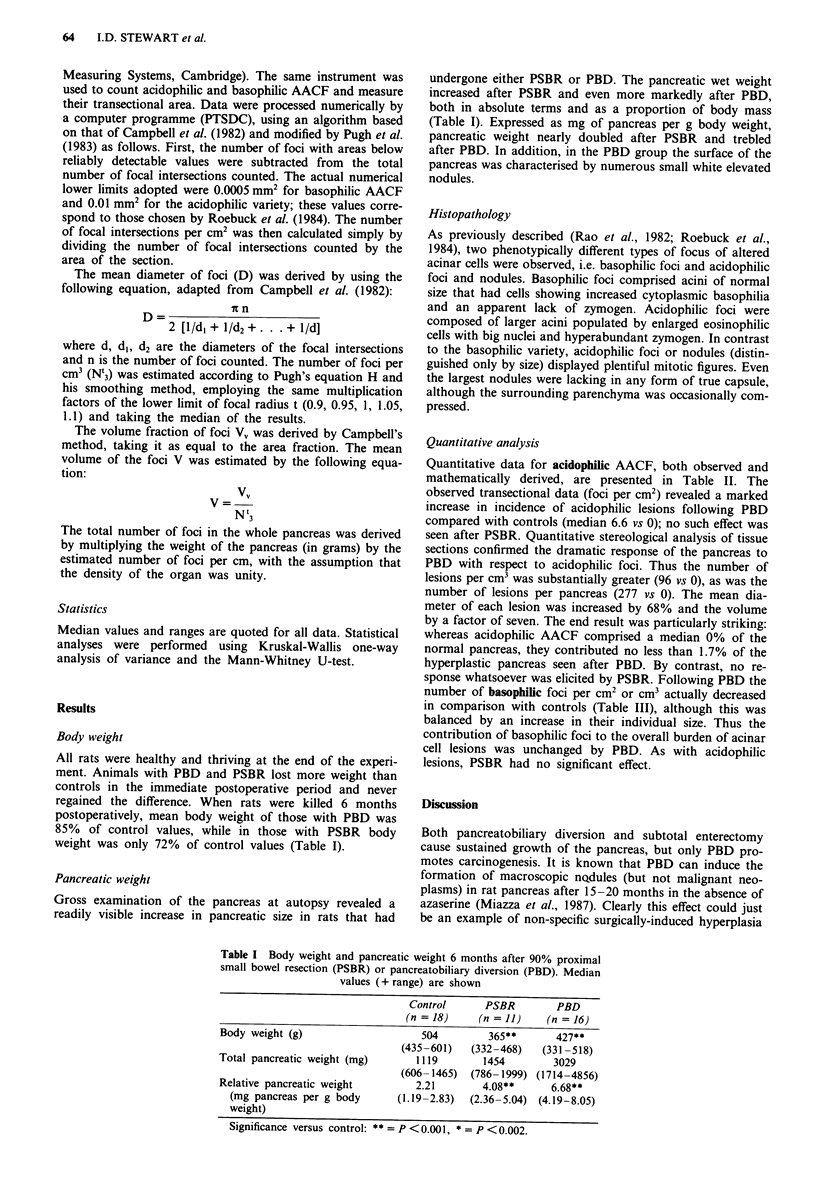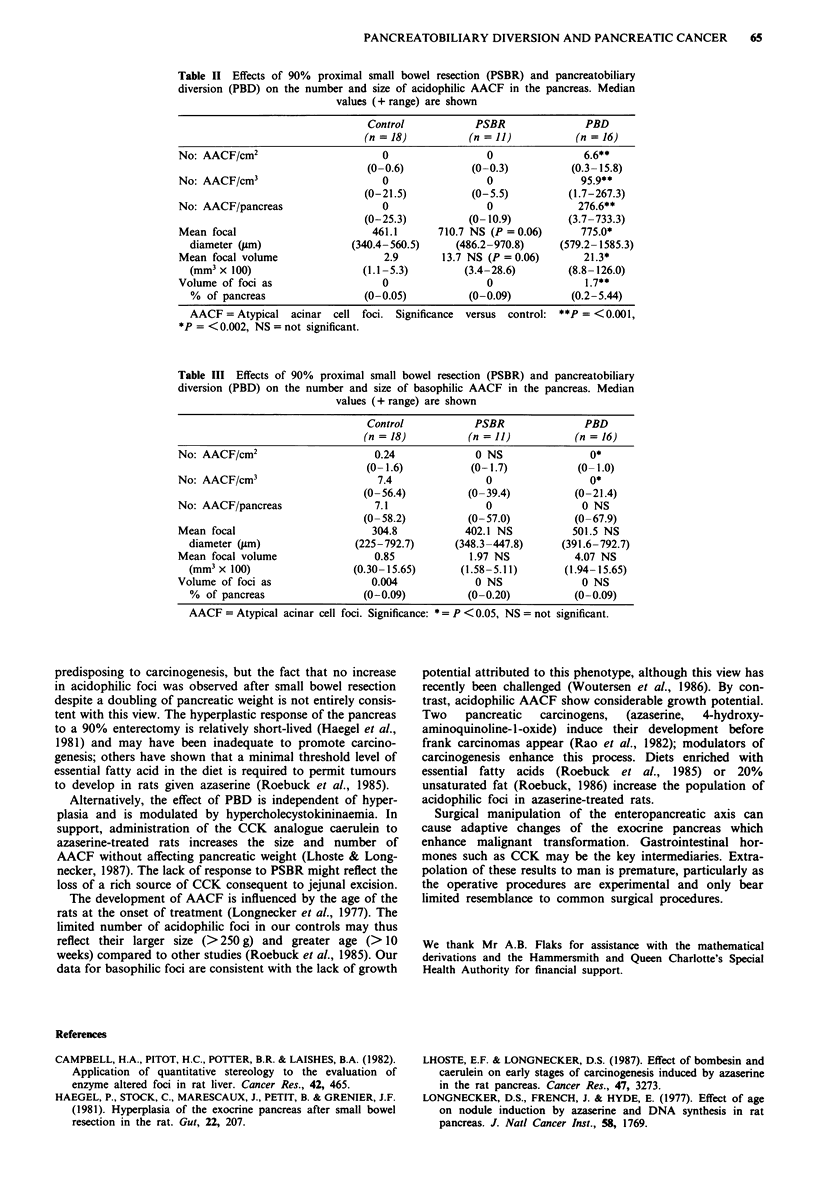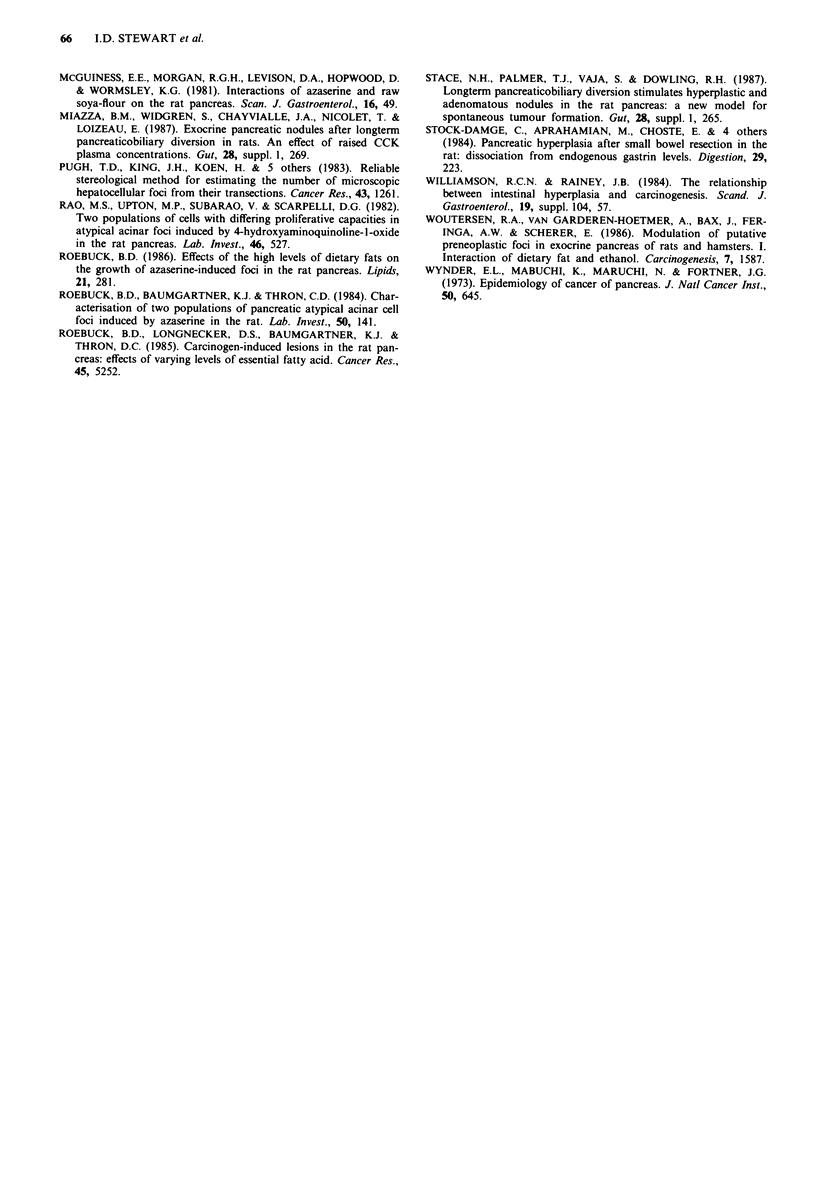# Pancreatobiliary diversion enhances experimental pancreatic carcinogenesis.

**DOI:** 10.1038/bjc.1991.13

**Published:** 1991-01

**Authors:** I. D. Stewart, B. Flaks, P. Watanapa, P. W. Davies, R. C. Williamson

**Affiliations:** Department of Surgery, Royal Postgraduate Medical School, Hammersmith Hospital, London, UK.

## Abstract

Since compensatory hyperplasia promotes experimental carcinogenesis in the gut, we tested the ability of two surgical models of pancreatic growth to promote pancreatic carcinogenesis. Male Wistar rats (n = 60) weighing 250-300 g underwent pancreatobiliary diversion (PBD), 90% small bowel resection (PSBR) or triple transection and reanastomosis of the small intestine (controls). Postoperatively, each group received azaserine (20 mg kg-1 wk-1 i.p.) for 6 weeks. Surviving rats were killed at 6 months, pancreatic wet weight was measured and histological sections were examined for atypical acinar cell foci (AACF), the putative precursor of carcinoma. Median relative pancreatic weight (mg pancreas/g body weight) was 2.20 for controls (n = 18), 4.08 for PSBR (n = 11) (P less than 0.001) and 6.86 for PBD (n = 16) (P less than 0.001). PSBR did not affect the development of acidophilic AACF, but PBD produced an enormous increase in their number per cm3 (median 96 vs. 0; P less than 0.001) and a 7-fold increase in their volume (P less than 0.001). Both operations cause pancreatic growth, but only PBD promotes carcinogenesis, possibly because of its unique hormonal effect.


Br. J. Cancer (1991), 63, 63–66 © Macmillan Press Ltd., 1991

### Pancreatobiliary diversion enhances experimental pancreatic carcinogenesis

I.D. Stewart[1], B. Flaks[2], P. Watanapa[1], P.W. Davies[1] & R.C.N. Williamson[1]

[1]*Department of Surgery, Royal Postgraduate Medical School, Hammersmith Hospital, Du Cane Road, London W12 0NN, UK; and* [2]*Department of Pathology, Medical School, University of Bristol, University Walk, Bristol BS8 8HW, UK.*

**Summary** Since compensatory hyperplasia promotes experimental carcinogenesis in the gut, we tested the ability of two surgical models of pancreatic growth to promote pancreatic carcinogenesis. Male Wistar rats ($n = 60$) weighing 250–300 g underwent pancreatobiliary diversion (PBD), 90% small bowel resection (PSBR) or triple transection and reanastomosis of the small intestine (controls). Postoperatively, each group received azaserine (20 mg kg$^{-1}$ wk$^{-1}$ i.p.) for 6 weeks. Surviving rats were killed at 6 months, pancreatic wet weight was measured and histological sections were examined for atypical acinar cell foci (AACF), the putative precursor of carcinoma. Median relative pancreatic weight (mg pancreas/g body weight) was 2.20 for controls ($n = 18$), 4.08 for PSBR ($n = 11$) ($P < 0.001$) and 6.86 for PBD ($n = 16$) ($P < 0.001$). PSBR did not affect the development of acidophilic AACF, but PBD produced an enormous increase in their number per cm$^3$ (median 96 *vs* 0; $P < 0.001$) and a 7-fold increase in their volume ($P < 0.001$). Both operations cause pancreatic growth, but only PBD promotes carcinogenesis, possibly because of its unique hormonal effect.

In the oesophagus, stomach and large intestine, carcinogenesis can be enhanced by luminal factors acting directly on the mucosa, but in the pancreas any such influence (particularly dietary) must be exerted indirectly through hormonal and/or neural connections, i.e. via the enteropancreatic axis. Presumably this type of pathway accounts for the correlation between a high-fat diet and pancreatic cancer mortality (Wynder *et al.*, 1973). In the stomach and colon, hyperplasia precedes and predisposes to neoplasia (Williamson & Rainey, 1984), and the same could be true for the pancreas. Feeding raw soya flour causes hyperplasia of the pancreas in rats and potentiates the carcinogenic effect of azaserine (McGuiness *et al.*, 1981).

Pancreatic growth can be stimulated by surgical operations. Thus transposition of a long segment of jejunum to lie between the pylorus and duodenal papilla will cause marked hyperplasia of rat pancreas within 7 days (Stace *et al.*, 1987). This effect may be mediated by an increased secretion of cholecystokinin (CCK) from the jejunum in the absence of pancreatic juice (Miazza *et al.*, 1987). Another surgical stimulus to pancreatic growth in rats is massive (90%) small bowel resection, though here the mechanism is unclear (Stock-Damge *et al.*, 1984): neither CCK nor gastrin appear to be involved.

The present study tests the hypothesis that surgical stimulation of pancreatic growth will enhance carcinogenesis. We have used pancreatobiliary diversion and subtotal enterectomy to manipulate the enteropancreatic axis and the formation of acidophilic AACF (atypical acinar cell foci) to indicate early malignant change (Roebuck *et al.*, 1984).

### Materials and methods

### *Animals*

Male Wistar rats weighing 250–300 g were housed five animals to a cage. Standard rat chow (Olac Ltd, Bicester, Oxon) and water were available *ad libitum*. Quarters were lighted in alternate 12 h light and dark cycles.

### *Operations*

Rats ($n = 60$) were divided into three equal groups. The first group (PSBR) had an extensive proximal small bowel resection; the proximal 90% of the jejunoileum was excised with an end-to-end anastomosis between the duodenojejunal flexure and the terminal 5 cm of ileum. The second group (PBD) received pancreatobiliary diversion as previously described (Stace *et al.*, 1987); the proximal half of the jejunoileum was transposed to lie between the pylorus and duodenal papilla. The third group (controls) had transection and resuture of the intestine at three sites: pylorus, duodenojejunal flexure, and mid small bowel. Surgical anastomoses were constructed with 6/0 silk sutures and ether anaesthesia was employed.

### *Carcinogen*

Azaserine was dissolved in 0.9% NaCl and was administered by weekly i.p. injection into each rat, starting immediately after operation and continuing for the next 6 weeks. The dosage regime was 20 mg kg$^{-1}$ wk$^{-1}$, giving a total dose of 120 mg kg$^{-1}$.

### *Mortality*

Eight rats died during or soon after operation from anaesthetic overdose or anastomotic leakage. Subsequently there were another seven premature deaths from intestinal obstruction or inanition (in animals with subtotal enterectomy). Yields of healthy survivors were as follows: Controls, $n = 18$; PBD, $n = 16$, PSBR $n = 11$.

### *Histology*

All rats were killed 6 months postoperatively. For morphological studies the pancreas was excised and carefully trimmed of all adherent fat, mesentery and lymph nodes. The wet weight of each gland was recorded before fixation in 10% buffered neutral formalin. Before immersion in the fixative solution, each pancreas was spread out on a piece of porous paper to ensure maximal transectional area for subsequent sectioning.

Histological sections (5 μm) were stained with haematoxylin and eosin and were examined by light microscopy. The atypical acinar cell foci (AACF) induced by azaserine were identified and classified as acidophilic or basophilic according to established criteria (Rao *et al.*, 1982).

### *Quantitative estimation of AACF*

The total area of exocrine pancreatic tissue was measured directly in a single histological section from each pancreas by means of a VIDS III video image analyser (Analytical

Correspondence: R.C.N. Williamson, Department of Surgery, Royal Postgraduate Medical School, Hammersmith Hospital, Du Cane Road, London W12 0NN, UK.
Received 23 February 1990; and in revised form 20 August 1990.

Measuring Systems, Cambridge). The same instrument was used to count acidophilic and basophilic AACF and measure their transectional area. Data were processed numerically by a computer programme (PTSDC), using an algorithm based on that of Campbell *et al.* (1982) and modified by Pugh *et al.* (1983) as follows. First, the number of foci with areas below reliably detectable values were subtracted from the total number of focal intersections counted. The actual numerical lower limits adopted were 0.0005 $mm^2$ for basophilic AACF and 0.01 $mm^2$ for the acidophilic variety; these values correspond to those chosen by Roebuck *et al.* (1984). The number of focal intersections per $cm^2$ was then calculated simply by dividing the number of focal intersections counted by the area of the section.

The mean diameter of foci (D) was derived by using the following equation, adapted from Campbell *et al.* (1982):

$$D = \frac{\pi n}{2\ [1/d_1 + 1/d_2 + \ldots + 1/d]}$$

where d, $d_1$, $d_2$ are the diameters of the focal intersections and n is the number of foci counted. The number of foci per $cm^3$ ($N'_3$) was estimated according to Pugh's equation H and his smoothing method, employing the same multiplication factors of the lower limit of focal radius t (0.9, 0.95, 1, 1.05, 1.1) and taking the median of the results.

The volume fraction of foci $V_v$ was derived by Campbell's method, taking it as equal to the area fraction. The mean volume of the foci V was estimated by the following equation:

$$V = \frac{V_v}{N'_3}$$

The total number of foci in the whole pancreas was derived by multiplying the weight of the pancreas (in grams) by the estimated number of foci per cm, with the assumption that the density of the organ was unity.

### *Statistics*

Median values and ranges are quoted for all data. Statistical analyses were performed using Kruskal-Wallis one-way analysis of variance and the Mann-Whitney U-test.

### Results

### *Body weight*

All rats were healthy and thriving at the end of the experiment. Animals with PBD and PSBR lost more weight than controls in the immediate postoperative period and never regained the difference. When rats were killed 6 months postoperatively, mean body weight of those with PBD was 85% of control values, while in those with PSBR body weight was only 72% of control values (Table I).

### *Pancreatic weight*

Gross examination of the pancreas at autopsy revealed a readily visible increase in pancreatic size in rats that had undergone either PSBR or PBD. The pancreatic wet weight increased after PSBR and even more markedly after PBD, both in absolute terms and as a proportion of body mass (Table I). Expressed as mg of pancreas per g body weight, pancreatic weight nearly doubled after PSBR and trebled after PBD. In addition, in the PBD group the surface of the pancreas was characterised by numerous small white elevated nodules.

### *Histopathology*

As previously described (Rao *et al.*, 1982; Roebuck *et al.*, 1984), two phenotypically different types of focus of altered acinar cells were observed, i.e. basophilic foci and acidophilic foci and nodules. Basophilic foci comprised acini of normal size that had cells showing increased cytoplasmic basophilia and an apparent lack of zymogen. Acidophilic foci were composed of larger acini populated by enlarged eosinophilic cells with big nuclei and hyperabundant zymogen. In contrast to the basophilic variety, acidophilic foci or nodules (distinguished only by size) displayed plentiful mitotic figures. Even the largest nodules were lacking in any form of true capsule, although the surrounding parenchyma was occasionally compressed.

### *Quantitative analysis*

Quantitative data for **acidophilic** AACF, both observed and mathematically derived, are presented in Table II. The observed transectional data (foci per $cm^2$) revealed a marked increase in incidence of acidophilic lesions following PBD compared with controls (median 6.6 *vs* 0); no such effect was seen after PSBR. Quantitative stereological analysis of tissue sections confirmed the dramatic response of the pancreas to PBD with respect to acidophilic foci. Thus the number of lesions per $cm^3$ was substantially greater (96 *vs* 0), as was the number of lesions per pancreas (277 *vs* 0). The mean diameter of each lesion was increased by 68% and the volume by a factor of seven. The end result was particularly striking: whereas acidophilic AACF comprised a median 0% of the normal pancreas, they contributed no less than 1.7% of the hyperplastic pancreas seen after PBD. By contrast, no response whatsoever was elicited by PSBR. Following PBD the number of **basophilic** foci per $cm^2$ or $cm^3$ actually decreased in comparison with controls (Table III), although this was balanced by an increase in their individual size. Thus the contribution of basophilic foci to the overall burden of acinar cell lesions was unchanged by PBD. As with acidophilic lesions, PSBR had no significant effect.

### Discussion

Both pancreatobiliary diversion and subtotal enterectomy cause sustained growth of the pancreas, but only PBD promotes carcinogenesis. It is known that PBD can induce the formation of macroscopic nodules (but not malignant neoplasms) in rat pancreas after 15–20 months in the absence of azaserine (Miazza *et al.*, 1987). Clearly this effect could just be an example of non-specific surgically-induced hyperplasia

**Table I** Body weight and pancreatic weight 6 months after 90% proximal small bowel resection (PSBR) or pancreatobiliary diversion (PBD). Median values (+ range) are shown

| | *Control (n = 18)* | *PSBR (n = 11)* | *PBD (n = 16)* |
|---|---|---|---|
| Body weight (g) | 504 | 365** | 427** |
| | (435–601) | (332–468) | (331–518) |
| Total pancreatic weight (mg) | 1119 | 1454 | 3029 |
| | (606–1465) | (786–1999) | (1714–4856) |
| Relative pancreatic weight | 2.21 | 4.08** | 6.68** |
| (mg pancreas per g body weight) | (1.19–2.83) | (2.36–5.04) | (4.19–8.05) |

Significance versus control: ** = $P < 0.001$, * = $P < 0.002$.

**Table II** Effects of 90% proximal small bowel resection (PSBR) and pancreatobiliary diversion (PBD) on the number and size of acidophilic AACF in the pancreas. Median values (+ range) are shown

| | *Control* (*n* = 18) | *PSBR* (*n* = 11) | *PBD* (*n* = 16) |
|---|---|---|---|
| No: AACF/cm$^2$ | 0 | 0 | 6.6** |
| | (0–0.6) | (0–0.3) | (0.3–15.8) |
| No: AACF/cm$^3$ | 0 | 0 | 95.9** |
| | (0–21.5) | (0–5.5) | (1.7–267.3) |
| No: AACF/pancreas | 0 | 0 | 276.6** |
| | (0–25.3) | (0–10.9) | (3.7–733.3) |
| Mean focal | 461.1 | 710.7 NS (*P* = 0.06) | 775.0* |
| diameter (μm) | (340.4–560.5) | (486.2–970.8) | (579.2–1585.3) |
| Mean focal volume | 2.9 | 13.7 NS (*P* = 0.06) | 21.3* |
| (mm$^3$ x 100) | (1.1–5.3) | (3.4–28.6) | (8.8–126.0) |
| Volume of foci as | 0 | 0 | 1.7** |
| % of pancreas | (0–0.05) | (0–0.09) | (0.2–5.44) |

AACF = Atypical acinar cell foci. Significance versus control: $^{**}P = <0.001$, $^{*}P = <0.002$, NS = not significant.

**Table III** Effects of 90% proximal small bowel resection (PSBR) and pancreatobiliary diversion (PBD) on the number and size of basophilic AACF in the pancreas. Median values (+ range) are shown

| | *Control* (*n* = 18) | *PSBR* (*n* = 11) | *PBD* (*n* = 16) |
|---|---|---|---|
| No: AACF/cm$^2$ | 0.24 | 0 NS | 0* |
| | (0–1.6) | (0–1.7) | (0–1.0) |
| No: AACF/cm$^3$ | 7.4 | 0 | 0* |
| | (0–56.4) | (0–39.4) | (0–21.4) |
| No: AACF/pancreas | 7.1 | 0 | 0 NS |
| | (0–58.2) | (0–57.0) | (0–67.9) |
| Mean focal | 304.8 | 402.1 NS | 501.5 NS |
| diameter (μm) | (225–792.7) | (348.3–447.8) | (391.6–792.7) |
| Mean focal volume | 0.85 | 1.97 NS | 4.07 NS |
| (mm$^3$ x 100) | (0.30–15.65) | (1.58–5.11) | (1.94–15.65) |
| Volume of foci as | 0.004 | 0 NS | 0 NS |
| % of pancreas | (0–0.09) | (0–0.20) | (0–0.09) |

AACF = Atypical acinar cell foci. Significance: * = $P < 0.05$, NS = not significant.

predisposing to carcinogenesis, but the fact that no increase in acidophilic foci was observed after small bowel resection despite a doubling of pancreatic weight is not entirely consistent with this view. The hyperplastic response of the pancreas to a 90% enterectomy is relatively short-lived (Haegel *et al.*, 1981) and may have been inadequate to promote carcinogenesis; others have shown that a minimal threshold level of essential fatty acid in the diet is required to permit tumours to develop in rats given azaserine (Roebuck *et al.*, 1985).

Alternatively, the effect of PBD is independent of hyperplasia and is modulated by hypercholecystokininaemia. In support, administration of the CCK analogue caerulein to azaserine-treated rats increases the size and number of AACF without affecting pancreatic weight (Lhoste & Longnecker, 1987). The lack of response to PSBR might reflect the loss of a rich source of CCK consequent to jejunal excision.

The development of AACF is influenced by the age of the rats at the onset of treatment (Longnecker *et al.*, 1977). The limited number of acidophilic foci in our controls may thus reflect their larger size (>250 g) and greater age (>10 weeks) compared to other studies (Roebuck *et al.*, 1985). Our data for basophilic foci are consistent with the lack of growth potential attributed to this phenotype, although this view has recently been challenged (Woutersen *et al.*, 1986). By contrast, acidophilic AACF show considerable growth potential. Two pancreatic carcinogens, (azaserine, 4-hydroxyaminoquinoline-1-oxide) induce their development before frank carcinomas appear (Rao *et al.*, 1982); modulators of carcinogenesis enhance this process. Diets enriched with essential fatty acids (Roebuck *et al.*, 1985) or 20% unsaturated fat (Roebuck, 1986) increase the population of acidophilic foci in azaserine-treated rats.

Surgical manipulation of the enteropancreatic axis can cause adaptive changes of the exocrine pancreas which enhance malignant transformation. Gastrointestinal hormones such as CCK may be the key intermediaries. Extrapolation of these results to man is premature, particularly as the operative procedures are experimental and only bear limited resemblance to common surgical procedures.

We thank Mr A.B. Flaks for assistance with the mathematical derivations and the Hammersmith and Queen Charlotte's Special Health Authority for financial support.

### References

CAMPBELL, H.A., PITOT, H.C., POTTER, B.R. & LAISHES, B.A. (1982). Application of quantitative stereology to the evaluation of enzyme altered foci in rat liver. *Cancer Res.*, **42**, 465.

HAEGEL, P., STOCK, C., MARESCAUX, J., PETIT, B. & GRENIER, J.F. (1981). Hyperplasia of the exocrine pancreas after small bowel resection in the rat. *Gut*, **22**, 207.

LHOSTE, E.F. & LONGNECKER, D.S. (1987). Effect of bombesin and caerulein on early stages of carcinogenesis induced by azaserine in the rat pancreas. *Cancer Res.*, **47**, 3273.

LONGNECKER, D.S., FRENCH, J. & HYDE, E. (1977). Effect of age on nodule induction by azaserine and DNA synthesis in rat pancreas. *J. Natl Cancer Inst.*, **58**, 1769.

MCGUINESS, E.E., MORGAN, R.G.H., LEVISON, D.A., HOPWOOD, D. & WORMSLEY, K.G. (1981). Interactions of azaserine and raw soya-flour on the rat pancreas. *Scan. J. Gastroenterol.*, **16,** 49.

MIAZZA, B.M., WIDGREN, S., CHAYVIALLE, J.A., NICOLET, T. & LOIZEAU, E. (1987). Exocrine pancreatic nodules after longterm pancreaticobiliary diversion in rats. An effect of raised CCK plasma concentrations. *Gut*, **28,** suppl. 1, 269.

PUGH, T.D., KING, J.H., KOEN, H. & 5 others (1983). Reliable stereological method for estimating the number of microscopic hepatocellular foci from their transections. *Cancer Res.*, **43,** 1261.

RAO, M.S., UPTON, M.P., SUBARAO, V. & SCARPELLI, D.G. (1982). Two populations of cells with differing proliferative capacities in atypical acinar foci induced by 4-hydroxyaminoquinoline-1-oxide in the rat pancreas. *Lab. Invest.*, **46,** 527.

ROEBUCK, B.D. (1986). Effects of the high levels of dietary fats on the growth of azaserine-induced foci in the rat pancreas. *Lipids*, **21,** 281.

ROEBUCK, B.D., BAUMGARTNER, K.J. & THRON, C.D. (1984). Characterisation of two populations of pancreatic atypical acinar cell foci induced by azaserine in the rat. *Lab. Invest.*, **50,** 141.

ROEBUCK, B.D., LONGNECKER, D.S., BAUMGARTNER, K.J. & THRON, D.C. (1985). Carcinogen-induced lesions in the rat pancreas: effects of varying levels of essential fatty acid. *Cancer Res.*, **45,** 5252.

STACE, N.H., PALMER, T.J., VAJA, S. & DOWLING, R.H. (1987). Longterm pancreaticobiliary diversion stimulates hyperplastic and adenomatous nodules in the rat pancreas: a new model for spontaneous tumour formation. *Gut*, **28,** suppl. 1, 265.

STOCK-DAMGE, C., APRAHAMIAN, M., CHOSTE, E. & 4 others (1984). Pancreatic hyperplasia after small bowel resection in the rat: dissociation from endogenous gastrin levels. *Digestion*, **29,** 223.

WILLIAMSON, R.C.N. & RAINEY, J.B. (1984). The relationship between intestinal hyperplasia and carcinogenesis. *Scand. J. Gastroenterol.*, **19,** suppl. 104, 57.

WOUTERSEN, R.A., VAN GARDEREN-HOETMER, A., BAX, J., FERINGA, A.W. & SCHERER, E. (1986). Modulation of putative preneoplastic foci in exocrine pancreas of rats and hamsters. I. Interaction of dietary fat and ethanol. *Carcinogenesis*, **7,** 1587.

WYNDER, E.L., MABUCHI, K., MARUCHI, N. & FORTNER, J.G. (1973). Epidemiology of cancer of pancreas. *J. Natl Cancer Inst.*, **50,** 645.